# Supplementary figures and images for: Defining the Roles of Pyruvate Oxidation, TCA Cycle, and Mannitol Metabolism in Methicillin-Resistant Staphylococcus aureus Catheter-Associated Urinary Tract Infection
Source: Microbiol Spectr. 2023 Jun 28;11(4):e05365-22. doi: 10.1128/spectrum.05365-22 (PMC10433999; doi:10.1128/spectrum.05365-22)

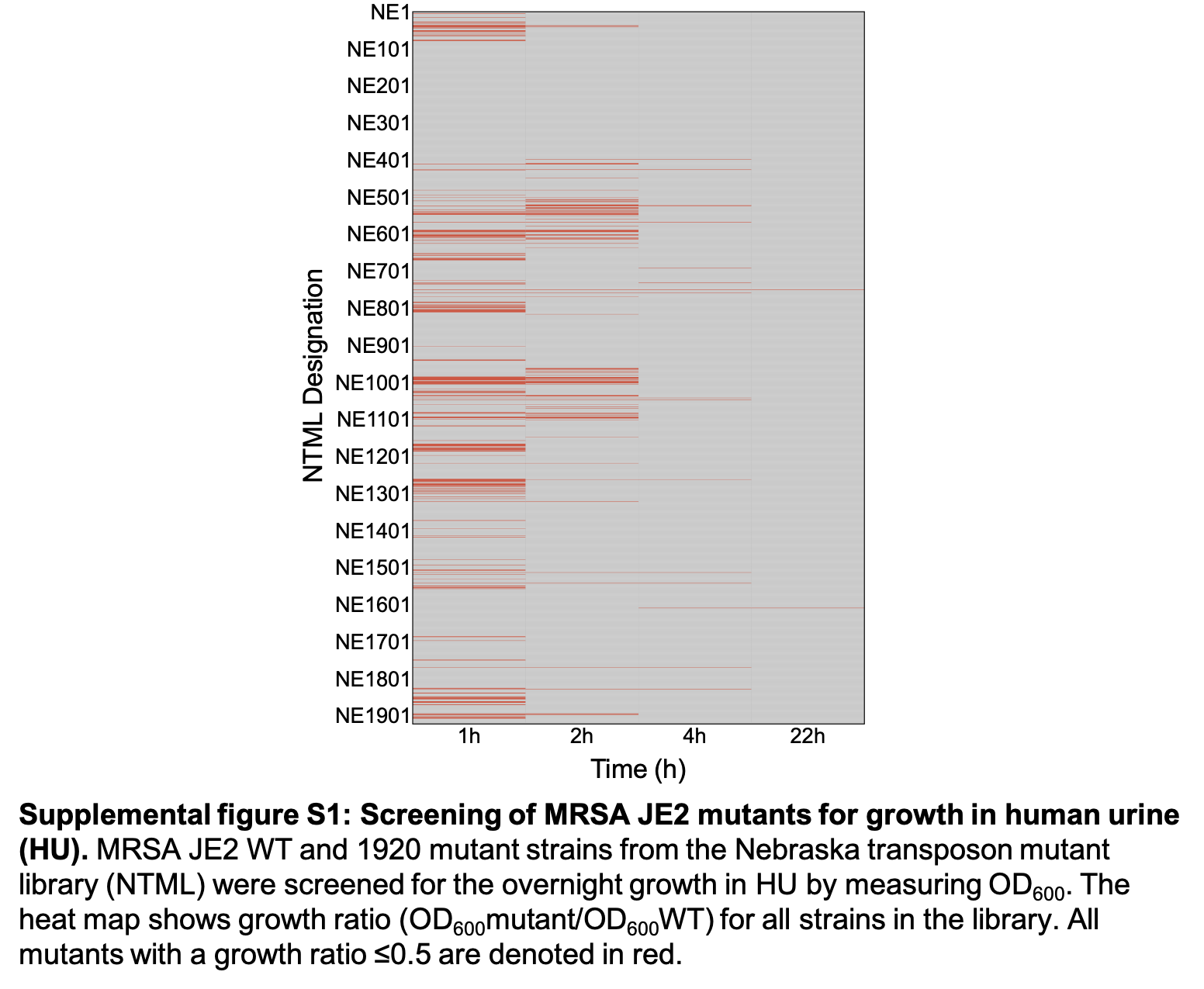

Supplement: Supplemental file 1 — Fig. S1. Download spectrum.05365-22-s0001.tif, TIF file, 7.6 MB [file spectrum.05365-22-s0001.tif]

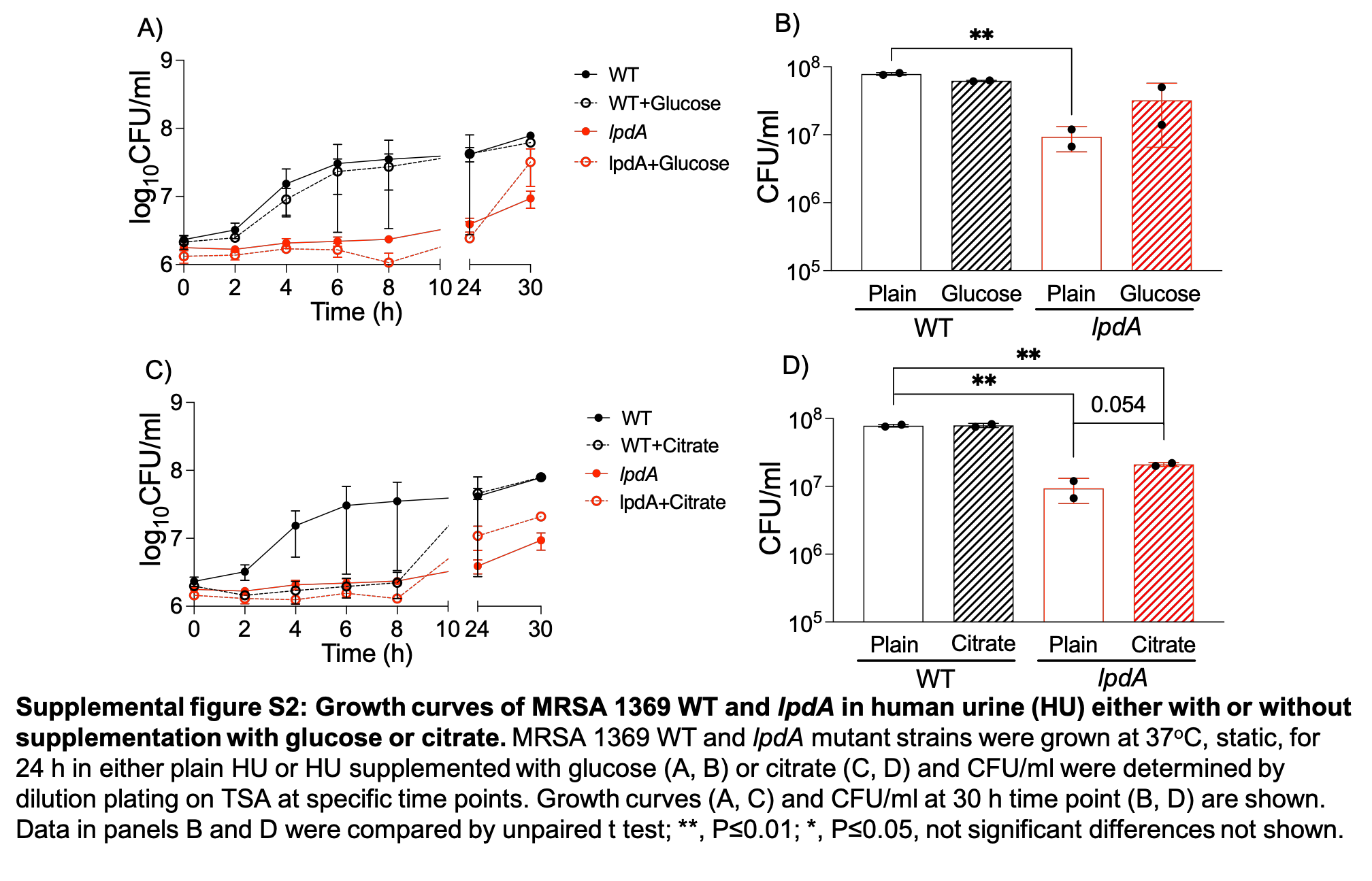

Supplement: Supplemental file 2 — Fig. S2. Download spectrum.05365-22-s0002.tif, TIF file, 10.0 MB [file spectrum.05365-22-s0002.tif]
